# The effect of eye movement desensitization on neurocognitive functioning compared to retrieval-only in PTSD patients: a randomized controlled trial

**DOI:** 10.1186/s12888-024-06420-9

**Published:** 2024-12-27

**Authors:** Eka Susanty, Marit Sijbrandij, Wilis Srisayekti, Yusep Suparman, Anja C. Huizink

**Affiliations:** 1 Faculty of Psychology, Universitas Jenderal Achmad Yani, Cimahi, Indonesia; 2 Department of Clinical, Neuro- and Developmental Psychology, Faculty of Behavioral and Movement Sciences, Vrije Universiteit Amsterdam, Amsterdam, the Netherlands; 3 Department of General and Experimental Psychology, Faculty of Psychology, Universitas Padjadjaran, Bandung, Indonesia; 4 Department of Statistics, Faculty of Mathematic and Natural Sciences, Universitas Padjadjaran, Bandung, Indonesia; 5 Department of Clinical, Neuro- and Developmental Psychology, Faculty of Behavioral and Movement Science, Vrije Universiteit Amsterdam, Van der Boechorststraat 7, Amsterdam, 1081 BT The Netherlands

**Keywords:** Posttraumatic stress disorder, Eye movement desensitization, Neurocognitive functioning, Learning memory, Attention, Working memory, Executive function, Information processing speed

## Abstract

**Background:**

There is robust evidence that posttraumatic stress disorder (PTSD) is associated with neurocognitive deficits, such as executive dysfunction or memory dysfunction. Eye Movement Desensitization and Reprocessing (EMDR) is an evidence-based treatment for PTSD, in which eye movements (EMs) are performed during traumatic memory retrieval. We examined whether Eye Movement Desensitization (EMD) improves neurocognitive functioning in PTSD patients, in comparison with a retrieval-only control condition without EMs.

**Methods:**

Adult patients with PTSD (*N* = 91) were randomized into EMD (*N* = 47) or retrieval-only (*N* = 44). Data were collected at baseline (T0), one-week post-treatment (T1), one-month follow-up (T2), and at three-month follow-up (T3). Outcome measures were the California Verbal Learning Test (CVLT), the Trail Making Test (TMT), and the Digit Span Subtest of the Wechsler Adult Intelligence Scale fourth edition (WAIS-IV). We conducted linear mixed model to analyse the main outcomes.

**Results:**

There was a main effect of time, indicating improvements for both the EMD and retrieval-only groups in CVLT scores, TMT A, TMT B and Digit Span score of WAIS-IV (Bonferroni-adjusted *p’s* < 0.001) from T0 to T3. There were no effects of group (*p* = .64) or group by time on CVLT total trial A (T3; *p = .*34), delay A (T3; *p =* .76), TMT A (T3; *p =* .61), TMT B (T3: *p =* .58), and Digit Span scores (T3; *p =* .78) of the WAIS-IV, indicating no significant differences between groups on any of the outcomes.

**Conclusion:**

Comparing EMD and retrieval-only did not show evidence for additive effects of EMs on the treatment of PTSD in terms of improvements in neurocognitive functioning. Thus, treatments based on retrieval of traumatic memories may be used to improve neurocognitive functioning in patients with PTSD.

**Clinical trial registration:**

The trial was registered 19/12/2017 at ClinicalTrials.gov, identifier [ISRCTN55239132].

**Supplementary Information:**

The online version contains supplementary material available at 10.1186/s12888-024-06420-9.

## Background

Posttraumatic stress disorder (PTSD) is a trauma and stressor-related disorder, which according to DSM-5 [1] is characterized by four categories: intrusion symptoms, persistent avoidance of stimuli associated with the trauma, negative alterations in cognitions and mood, and alterations in arousal and reactivity. Previous studies in PTSD patients showed that structural and functional abnormalities have been found in the prefrontal cortex, amygdala, and hippocampus [2]. These brain regions are responsible for cognitive and emotional processing (e.g [3, 4].

Alterations in cognitive function are a category of PTSD symptoms that are associated with neurocognitive problems, as per the DSM-5 (e.g., problems with controlled traumatic memories, hypervigilance to threat, and difficulty sustaining attention). Several reviews [5] and meta-analyses [6–9] have shown robust evidence that impaired neurocognitive function is part of PTSD symptomatology, namely decreased learning and verbal memory (e.g [10]). episodic memory [8, 11], attention [5, 12], executive function (e.g [7]. inhibition [13–15], switching/shifting [16, 17], updating working memory [18], and information processing speed [19, 20]. A meta-analysis also reported that adults with PTSD suffer from mild to moderate impairments in executive function tasks [7]. Furthermore, a recent meta-analyses of neurocognitive deficits in PTSD revealed impairments in episodic memory beyond traumatic experiences, which were particularly evident on verbal memory tests [8].

Despite numerous studies that have focused on PTSD and neurocognitive dysfunction, a limited number of studies have examined whether neurocognitive outcomes improve following PTSD treatment. A recent systematic review comprised four non-randomized studies and five randomized controlled trials (RCTs) found no significant effects of psychological treatments for PTSD on executive function, working memory and information processing, but a significant improvement in memory [21]. There have been endeavours to enhance neurocognitive function in PTSD patients using neurofeedback [22] or computer-based interventions [23, 24]. However, those studies have not provided consistent results. Furthermore, the positive effect of treatment on neurocognitive outcomes suggests that during treatment, patients may learn to compensate their neurocognitive difficulties [25]. For instance, in trauma-focused therapy, the patient’s improved capacity to narrate traumatic memories, encode and recall the content of traumatic memory, or re-contextualize memories may improve their verbal memory deficits [26, 27]. Thus, there exists limited evidence suggesting that certain PTSD treatments can have a positive effect on particularly memory deficits that are often found in PTSD patients.

Eye Movement Desensitization and Reprocessing (EMDR) is recognized as a first-line therapy recommendation for individuals with PTSD, with empirical support [28, 29]. Research has demonstrated that EMDR is more effective than both waitlist conditions and standard care in treating PTSD among adults [30–32]. However, the American Psychological Association (APA) [33] considers EMDR a conditionally recommended treatment for adult PTSD, citing limited evidence supporting its efficacy for certain populations, particularly those with combat-related PTSD [34].

According to the working memory theory of EMDR, eye movements are considered to be essential to the effects of EMDR, because they tax the working memory during memory retrieval, which has a limited capacity [35]. By concentrating on bilateral EMs while simultaneously retaining the emotion in mind, the reconsolidation of traumatic memories may be disrupted, resulting in a decline in the episodic quality of the memory and thus decreasing PTSD symptoms [36, 37]. Both experimental studies in the laboratory with healthy participants and studies in clinical samples have supported the working memory theory as an explanation for the effect of EMs in EMDR. A meta-analysis investigating the role of EMs within therapeutic contexts revealed moderate effects of EMs in reducing PTSD symptoms or subjective units of distress compared to treatment without EMs [38]. Similarly, another meta-analysis on analog lab studies examining the additive benefits of EMs in retrieving aversive memories showed a superior effect of EMs on vividness and emotionality of these memories [39].

However, some researchers have suggested that EMDR largely promotes change through exposure to the traumatic memory [40]. Indeed, similar to other trauma-focused interventions such as exposure therapy, EMDR involves the recollection of traumatic memories and exploration of their related emotions, meanings, and physical sensations [41]. Clinical studies have supported this notion, with many of them not finding a difference in effectiveness between EMDR and exposure therapy [42–44].

Although the efficacy of EMDR in alleviating PTSD symptoms has been demonstrated, studies that assess its impact on neurocognitive functioning are scarce. To the best of our knowledge, no dismantling study has investigated the effect of EMs on neurocognitive function [39]. This dismantling study focuses on Eye Movement Desensitization (EMD), the initial component of EMDR in improving neurocognitive functioning. We assessed numerous neurocognitive functions that have been proven to experience decline in individuals with PTSD [6]. PTSD can be classified as a disorder of memory and decreased attention [45–47], due to the fact that patients experience vivid and emotional memories that are not well consolidated into long-term memory [48]. In accordance with recent discoveries in the neuroscience of PTSD that have proposed a more significant pathophysiological role for prefrontal cortex [6, 49], we also focus on executive function [12, 50] and information processing [6, 51, 52].

The objective of this study was to examine the effectiveness of EMD in improving neurocognitive functioning among PTSD patients, as compared to retrieval-only, involving the same process as EMD, but without the use of EMs. We also investigated whether changes in neurocognitive functioning persisted after one month or three months following treatment. We hypothesized that EMDR participants would show more improvements in neurocognitive functioning at one month (T2) and three months (T3) following treatment compared to retrieval-only participants. In addition, we explored whether there were differences in neurocognitive outcomes between recovered (score of the PTSD Checklist for DSM-5 (PCL-5) < 33 after treatment) versus non-recovered patients and responders (a more than 50% decrease in mean PCL-5 scores after treatment) versus non-responders in both conditions. Finally, we hypothesized that recovered patients and responders would show better neurocognitive outcomes than non-recovered patients or non-responders, respectively after treatment. The current study was part of larger project on the effects of EMs on a stress-related measures, symptoms of PTSD and depression, and neurocognitive outcomes [53].

## Method

### Design

We conducted an RCT in which EMD was compared with a retrieval-only condition as control group. Assessments were scheduled at one-week post-treatment (T1), at one month (T2) and three months (T3) following the conclusion of treatment. The Health Research Ethics Committee of the Medical Faculty of Universitas Padjadjaran (KEPK-FK Unpad) granted approval for the study on 2 July 2018. The RCT has been registered prospectively [54] and the study protocol has been described elsewhere [53]. This paper is one of the three papers based on the RCT, each focusing on these different domains. A first domain of outcomes described in Susanty et al. (2024) [55] was heart rate (HR), pre-ejection period (PEP), cortisol [55]. A second domain of outcomes were the mental health outcomes, including symptoms of PTSD, assessed with the PTSD Checklist for DSM-5 (PCL-5), stress assessed with Perceived Stress Scale (PSS), depression and anxiety assessed with the Hopkins Symptoms Checklist-25 (HSCL-25), and the Brief Version of World Health Organization Quality of Life (WHOQOL) [56].

### Participants

The process of recruiting participants took place between April 1, 2019 and December 31, 2020. Participants were treatment-seeking outpatients with a PTSD diagnosis according to DSM-5 [1] who were recruited from three public psychological services: the “Pulih” clinic in Jakarta, the “Unisba psychology service” in Bandung and the “Unjani crisis center” in Cimahi, Indonesia. The following were the criteria for inclusion: (1) a DSM-5 diagnosis of PTSD as determined by the Structured Clinical Interview for DSM-5 Disorders (SCID-5); and (2) an age of at least 18 years. The criteria for exclusion were: (1) a current or past psychotic disorder; (2) a current substance use disorder; (3) acute suicidality as measured by the SCID-5; or (4) a current organic disorder; (5) in current psychotherapy or current medication. We also excluded patients who had previously taken psychotropic medication or recreational substances that may impacted treatment.

The power calculations were based on the assumption of a significant difference between the two treatment arms on heart rate variability (HRV), a stress measure outcome which served as the primary outcome of the overall project (see [53]. HRV results will be described elsewhere. For power calculations to indicate a difference with an expected effect size of *d* = 0.4 (see [38], a minimum sample size of 41 participants per group was recommended. To account for a possible 25% dropout rate at follow-up, the target number of participants was 110 (55 per group). We halted recruitment after including 90% of the original participants in our follow-up assessment amidst the challenges of the COVID-19 pandemic.

### Study procedures

The participants were informed by a bachelor-level trained assessor of the study’s objectives and asked for oral and written informed consent. At baseline, neurocognitive tests administered included the Trail Making Test (TMT) [57], the California Learning Verbal Test (CLVT; [58], and Digit Span Subtest of the Wechsler Adult Intelligence Scale fourth edition (WAIS-IV) [59].

After the baseline assessment, participants were randomized by a blinded assessor using Castor EDC [60] on a 1:1 ratio via block randomization (block sizes 4, 6, and 8) into one of two conditions: (1) EMD or (2) retrieval-only control. The duration between T0 and the initial intervention session was approximately one week. At post-treatment (T1) after the final treatment session, TMT, CVLT, and Digit Span were re-administered, and subsequently at one month (T2) and three months (T3) after the final session. The assessors were blinded to treatment allocation.

### Interventions

The procedures of EMD [61] thus comprised the subsequent procedures (for a comprehensive description, See also [53]: (1) *Clinical history and treatment planning*: gathering a medical history and developing a treatment plan; (2) *Preparation*: explanation of EMD process and its benefits; (3) *Assessment* of the target visual image, the patient rates the intensity of the negative emotions on a Subjective Units of Distress (SUD) scale ranging from 0 (indicating no disturbance or neutrality) to 10 (indicating the highest level of distress); (4) *Desensitization* recall of the target traumatic memory while the participant focuses their eyes on the therapist’s finger that rotates from left to right and back in the participant’s visual field for 24 cycles and 5 to 8 times. Following this, participants were directed to perform a body scan until any feelings of tension vanished; (5) *Closure*; a review is conducted of the relaxation exercises and stabilization techniques. Session 2–4 starts with a reassessment of the patient’s progress and SUD scores for target events. We decided to omit the installation phase (performing EMs while retrieving a positive cognition or image) from the original EMDR procedure in both study groups because it has been suggested to be counterproductive and may unintentionally render positive images less vivid and positivistic [35].

EMD was given for 4 to a maximum of 6 sessions, with each session lasting 60–90 min [62]. EMD was terminated when the SUDs reached 0 or 1 for all target memories during at least 4 sessions, or when a total number of 6 sessions was reached. The entire therapy process was video recorded with the patient’s consent. All sessions in all treatment condition were delivered by eight psychotherapists with at least one year of experience in treating PTSD patients. Therapists were provided training on how to administer the EMD protocol properly before the start of the study. Therapists were supervised weekly by an accredited EMDR supervisor throughout the study.

#### Retrieval-only condition (control)

The control participants were administered the identical treatment as the EMD participants, with the exception that no EMs were performed during phase 4: desensitization, when the traumatic memory was retrieved.

### Measures

The CVLT is a list learning task that assesses the ability to encode and recall new information after a short delay free recall [63]. Total free recall five trials in list A are referred to a measure of encoding performance. After words in the B list (immediate free recall) are given, participants are asked immediately to recall words in the A list (short delay free recall) [64]. In the current study, we analyzed the CVLT scores by focusing on the short-delay recall test without including long-delay recall trials. We employed a cutoff trial A (total score less than 43) and delay A (score less than 8) to define “impairment” based on a previous study that included a sample of PTSD patients [65]. The CVLT second edition has been translated and adapted for use in Indonesia and showed good psychometric properties [66].

The TMT part A was used to assess information processing speed and attention; part B was used to assess executive task switching and divided attention [57]. The TMT has high interrater reliability on both parts [67].The score for each part indicates how long it will take to complete the task [68]. A cut-off period of 300 s is commonly utilized to end test administration and is hence the typical maximum score [69]. The time to complete part A and part B both represent mental speed, with part A focuses on information processing and part B focuses on cognitive flexibility. In a sample of PTSD patients, the clinical threshold for the raw scores of the TMT A and TMT B were > 48 s and > 117 s, respectively [65].

The Digit Span subtest of standard WAIS-IV was used in the present study. The Digit Span is a three-part test that involves forward, backward and sequencing digit span [70]. Each part of the Digit Span subtest consists of 8 items and scores range between 2 and 24. The WAIS-IV Digit Span was used to assess attention, encoding and auditory processing (forward), working memory, transformation of information (backward) and mental manipulation of information (sequence) [71]. We employed the age-corrected criterion for Digit Span, which is less than 6, to define cognitively impaired individuals in the mixed clinical sample (of 49% were cognitively impaired) [70].

The SCID-5 is a semi-structured interview to diagnose DSM-5 Axis I disorders [72]. During screening, the Trauma and Stressor-Related Disorder to diagnose PTSD, Psychotic and Associated Symptoms, and Substance Use Disorders Modules were implemented using the Indonesian version of the SCID-5 [73].

The 20-item PCL-5 self-report questionnaire addresses DSM-5 PTSD symptoms from the past month. Participants rate their PTSD symptoms from 0 to 4 (“not at all (0)” to “extremely [4]”). The total severity score ranges from 0 to 80, with higher scores indicating more severe symptoms. DSM-5 symptom cluster severity scores are generated by adding up the item scores per cluster, namely “intrusion” (items 1–5), “avoidance” (items 6–7), “negative in cognition and mood” (items 8–14), and “arousal and reactivity” (items 15–20). After translating and validating the Indonesian PCL based on DSM-5, the study showed strong internal consistency (Cronbach’s alpha = 0.89).

### Statistical analysis

Baseline clinical and sociodemographic characteristics were compared between treatment conditions and between patients who dropped out and those who did not drop out at T1, T2, and T3. We used Fisher’s exact tests and independent-sample t-tests in SPSS version 26. For the categorical variables, the results were shown as percentages and numbers. For the continuous variables, the results were shown as means, standard deviations, and minimum and maximum values.

We used linear mixed models in R versions 4.4.1 and the “nlme” package (Linear and Nonlinear Mixed Effects Models. R package version 4.4.1), with a random effects model to compare the effect of EMD versus retrieval-only on neurocognitive outcomes. Time, condition (EMD versus retrieval-only), and time by condition were all included. All outcomes are reported as unstandardized regression coefficients. A time by condition term represented the effect of EMD and retrieval-only interventions on the outcome variables over time in all analyses. In the overall project, a total number of 11 statistical tests will be conducted on all outcomes (for an overview see [53]). To correct for multiple testing at post-hoc testing, we used applied Bonferroni correction considering 11 tests (alpha level was 0.05/11 = 0.005). All randomized participants were included in the intent to treat (ITT) sample. The regression method was employed to impute missing data for participants who failed to complete the T2 and/or T3 assessments. We conducted post-hoc analyses (Bonferroni correction) for both ITT and completer (per protocol). For the per-protocol analysis, participants who completed a minimum of four sessions were included.

Subsequently, we also conducted exploratory analyses with t-tests to examine differences in neurocognitive outcomes between recovered as compared to non-recovered patients and responders as compared to non-responders. A recovered patient is a patient with a score of PCL-5 lower than 33 after treatment and participant in either the EMD or the control condition [74]. A responder is a patient with a more than 50% decrease in mean PCL-5 scores after either EMD or retrieval-only treatment [75]. Furthermore, we conducted a Reliable Change Index (RCI) analysis using adjusted RCI based on regression to ascertain whether the changes in scores on repeat tests reflect actual improvement or practice effects [76].

## Results

### Participants

**Fig. 1:**
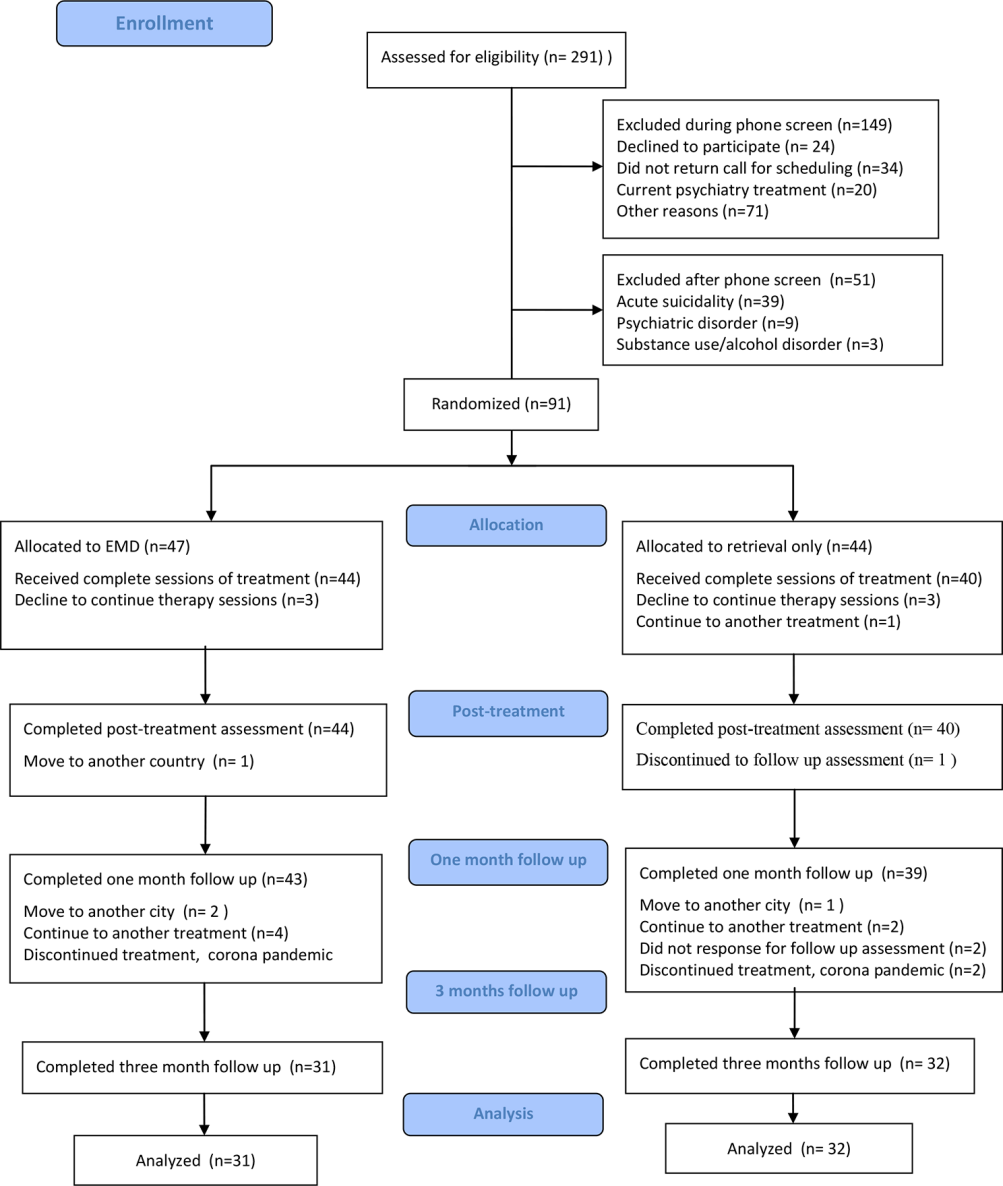
Consort flow diagram

Out of the 291 individuals who were approached, 91 (31.3%) consented to take part in the study. A remainder of 84 participants performed the T1 assessment. Participant enrolment and flow during the course of the study is presented in Fig. 1. For the retrieval-only group, the follow-up rates were 90.9% (40/44) at T1, 88.6% (39/44) at T2, and 72.7.% (32/44) at T3. For the EMD group, the follow-up rates were 93.6% (44/47) at T1, 91.5% (43/47) at T2, and 66.0% (31/47) at T3. The participant dropout rate was relatively low at 7.7% (84/91), indicating that the sample size of 84 patients was met at posttreatment (T1) to detect statistical differences. Overall, the percentage of participants following the completed assessment from T0 to T3 was 69.2% (63/91). Of the 91 participants, 79 participants completed all six EMD sessions. Twelve participants did not complete all six EMD sessions (9 completed five and 3 completed four sessions).

Table 1 shows the baseline demographic and clinical characteristics for each group. At baseline, there were no significant differences in sociodemographic, PTSD symptom, or neurocognitive measures between the 47 EMD and 44 retrieval-only participants (see Table 1). This means randomization works well. The majority of participants were female, with 89.4% (42/47) of the EMD and 93.2% (41/44) of the retrieval-only group. In addition, 57.4% (27/47) of the EMD and 56.8% (25/44) of the retrieval-only group had experienced domestic violence. In terms of baseline demographic and clinical characteristics, patients who dropped out at T1, T2, and T3 did not differ significantly from those who completed the assessments. Furthermore, PCL-5 total scores decreased significantly from baseline to T3 in both groups (*p* < .001). Post-hoc testing revealed that PCL-5 total scores were lower at T1, T2, and T3 compared to T0 in both EMD and retrieval groups (*p* < .005) (see [56]).

**Table 1:** Baseline characteristics of participants

Variable	Total *N* = 91	Retrieval only (*n* = 44)	EMD (*n* = 47)	Fisher’s exact or t	*P*-value*
**Age, mean (SD)**	25.43 (6.23)	24.66 (5.51)	26.15 (6.81)	-1.14	0.26
**Sex, n (%)**				0.42	0.72
Male	8 (8.8)	3 (6.8)	5 (10.6)		
Female	83 (91.2)	41 (93.2)	42 (89.4)		
**Education, n (%)**				3.76	0.29
High school	33 (36.6)	18 (40.9)	15 (31.9)		
College	5 (5.5)	4 (9.1)	1 (2.1)		
Bachelor	49 (53.8)	21 (47.7)	28 (59.6)		
Master	4 (4.4)	1 (2.3)	3 (6.4)		
**Work, n (%)**				0.78	0.76
Unemployed	47 (51.6)	21 (47.7)	26 (55.3)		
Public sector	2 (2.2)	1 (2.3)	1 (2.1)		
Private sector	42 (46.2)	22 (50.0)	20 (42.6)		
**Marital status, n (%)**				1.04	0.9
Unmarried	69 (75.8)	33 (75)	36 (76.6)		
Married	21 (23.1)	10 (22.7)	11 (23.4)		
Divorced	1 (1.1)	1 (2.3)	0 (0)		
**Trauma type, n (%)**				1.28	0.58
Domestic violence	52 (57.1)	25 (56.8)	27 (57.4)		
Sexual abuse	13 (14.3)	8 (18.2)	5 (10.6)		
Other	26 (28.6)	11 (25)	15 (31.9)		
**PCL-5, mean (SD)**					
PCL-5 total	58.41 (9.36)	57.93 (9.07)	58.85 (9.68)	-0.47	0.64
Intrusive Avoidance	15.46 (2.93)	15.48 (3.05) 5.86 (1.73)	14.45 (2.85) 6.30 (1.49)	-0.05	0.96 0.20
	6.09 (1.62)			-1.28	
Thinking and Mood	20.11 (4.59)	19.77 (4.69)	20.43 (4.52)	-0.67	0.50
Arousal and reactivity	16.75 (3.52)	16.82 (3.16)	16.68 (3.87)	− 0.19	0.85
*Neurocognitive functioning* **CVLT**					
CVLT total CVLT trial A CVLT trial B CVLT delay A **TMT**	55.62 (9.07) 49.57 (8.03) 6.04 (1.78) 21.39 (5.59)	55.32 (9.96) 49.50 (8.89) 5.82 (1.54) 20.98 (5.97)	55.91 (8.21) 49.64 (7.20) 6.27 (1.98) 21.80 (5.24)	-0.31 -0.84 -1.19 -0.69	0.76 0.93 0.23 0.49
TMT A TMT B **Digit Span (WAIS-V)** Digit Span total Digit span forwarded Digit span backward Digit span sequence	50.21 (32.66) 78.80 (36.97) 16.48 (2.40) 5.94 (1.21) 4.53 (0.99) 6.01 (1.29)	50.11 (37.54) 82.25 (43.49) 16.36 (2.61) 5.77 (2.20) 4.57 (1.04) 6.02 (1.42)	50.31 (27.51) 75.42 (29.34) 16.60 (2.20) 6.11 (2.21) 4.49 (0.94) 6.00 (1.17)	0.54 0.20 -0.46 -1.33 0.37 0.09	0.97 0.39 0.65 0.19 0.71 0.93

Notes: *Significance, *p* < .005

Chi-square test for nominal variables and independent samples t tests for continuous variables

EMD = Eye Movement Desensitization

CVLT; the California Verbal Learning, PTSD; Posttraumatic Stress Disorder, TMT; Trail Making Test, WAIS-V; Wechsler Adult Intelligence Scale for Diagnostic Statistical Manual –V

### Treatment effects

#### Verbal learning (CVLT)

There was an overall effect of time for trial A total score. The findings revealed that the total scores of trial A increased significantly from baseline to T3 for both groups (T3; *p* < .001). The post-hoc test showed that trial A scores for both groups were greater at T1, T2, and T3 than at T0 (Bonferroni-adjusted *p* < .001). Trial A scores were not affected by group (*p* = .70) or group by time interaction (T3; *p* = .34), according to our findings. Thus, Linear Mixed Models (LMM) revealed that there were no significant differences in trial A total scores at T1 between the EMD and retrieval-only groups (M [SE] 57.40 [2.03] vs. 57.20 [2.05], *p* = .93 95% CI -3.52 to 3.20) or at T2 (M [SE] 61.30 [2.03] vs. 60.60 [2.05], *p* = .68, 95% CI -4.05 to 3.20) or at T3 (M [SE] 64.90 [2.03] vs. 62.60 [2.05], *p* = .18, -5.66 to 1.06). Summary statistics from LMM intention to-treat analysis for CVLT, TMT and Digit Span are presented in Table 2. Per-protocol analysis for CVLT indicated similar results (appendix D). Appendix B contains the ITT analysis of the estimates for the effects of group, time, and group by time interactions on CVLT, TMT, and Digit Span. Analyses of estimates conducted in accordance with protocol for the effects of group, time, and group by time interaction on CVLT, TMT, and Digit Span yielded comparable outcomes (see Appendix C).

**Table 2:** Summary statistics and results from mixed-model analysis of CVLT, TMT and Digit Span (intention-to-treat sample, *N* = 91)

Outcomes	Measurement time	Mean (Standard Error)				Mean difference (95% confidence interval)			*P-*values
		Retrieval only		EMD					
Total trial A (learning)	T1	57.20 (2.05)	57.40 (2.03)				-0.16 (-3.52 to 3.20)	0.93	
	T2	60.60 (2.05)	61.30 (2.03)				-0.69 (-4.05 to 2.67)	0.68	
	T3	62.60 (2.05)	64.90 (2.03)				-2.30 (-5.66 to 1.06)	0.18	
Trial B (distractor)	T1	6.60 (0.42)	6.33 (0.41)				0.27 (-0.45 to 0.99)	0.46	
	T2	6.74 (0.42)	6.58 (0.41)				0.16 (-0.56 to 0.88)	0.66	
	T3	7.24 (0.42)	7.23 (0.41)				0.01 (-0.72 to 0.73)	0.98	
short delay recall trial A	T1	25.60 (1.03)	25.20 (1.01)				0.39 (-1.42 to 2.20)	0.67	
	T2	26.00 (1.03)	27.00 (1.01)				-1.01 (-2.82 to 0.79)	0.27	
	T3	26.50 (1.03)	27.50 (1.01)				-1.00 (-2.81 to 0.80)	0.27	
TMT A	T1	40.40 (5.73)	37.90 (5.65)				2.49 (-7.06 to 12.03)	0.61	
	T2	35.00 (5.73)	32.10 (5.65)				2.87 (-6.68 to 12.41)	0.55	
	T3	30.00 (5.73)	27.00 (5.65)				3.00 (-6.54 to 12.54)	0.53	
TMT B	T1	68.00 (8.25)	66.70 (8.14)				1.31 (-11.97 to 14.59)	0.84	
	T2	65.60 (8.25)	56.50 (8.14)				9.09 (-4.19 to 22.37)	0.18	
	T3	52.20 (8.25)	47.50 (8.14)				4.66 (-8.62 to 17.94)	0.49	
Digit span total	T1	18.40 (0.73)	17.60 (0.72)				0.83 (-0.30 to 1.97)	0.15	
	T2	18.70 (0.73)	18.30 (0.72)				-0.38(-0.76 to 1.52)	0.51	
	T3	18.20 (0.73)	18.60 (0.72)				-0.42 (-1.55 to 0.72)	0.47	
Digit span forward	T1	6.23 (0.29)	6.17 (0.28)				0.05 (-0.43 to 0.54)	0.83	
	T2	6.36 (0.29)	6.26 (0.28)				0.11 (-0.38 to 0.59)	0.67	
	T3	6.20 (0.29)	6.13 (0.28)				0.07 (-0.41 to 0.56)	0.76	
Digit span backward	T1	4.96 (0.28)	4.63 (0.27)				0.07 (-0.39 to 0.52)	0.15	
	T2	4.91 (0.28)	5.05 (0.27)				0.33 (-0.13 to 0.79)	0.54	
	T3	4.87 (0.28)	4.86 (0.27)				-0.14 (-0.60 to 0.32)	0.98	
Digit span sequence	T1	6.96 (0.34)	6.63 (0.33)				-0.33 (-0.22 to 0.89)	0.24	
	T2	7.25 (0.34)	6.88 (0.33)				0.37 (-0.18 to 0.93)	0.19	
	T3	7.30 (0.34)	7.31 (0.33)				-0.01 (-0.56 to 0.55)	0.98	

Note: * Bonferroni correction-significant *p* < .005

EMD = Eye Movement Desensitization

CVLT; the California Verbal Learning, PTSD; Posttraumatic Stress Disorder, TMT; Trail Making Test

Similarly, we found significant an overall effect of time for short delay A scores in both groups (appendix B). The results indicated a significant improvement in short delay A score from baseline to post-treatment at 3-months (*p* < .001) in both groups. Post-hoc analysis revealed that delay A scores were greater at T1, T2, and T3 than T0 for both groups (Bonferroni-adjusted *p* < .001). However, there was no effect of group (*p* = .46) nor group by time interaction (T3; *p* = .76) on delay A scores. The LMM analysis showed no statistically significant differences in delay A total scores at T1 between the EMD and retrieval-only groups (M [SE] 25.20 [1.01 vs. 25.60 [1.03], *p = .*67 95% CI -1.42 to 2.20) or at T2 (M [SE] 27.00 [1.01 vs. 27.00 [1.03], *p* − .27 95% CI -2.82 to 0.79) or T3 (M [SE] 27.50 [1.01] vs. 26.50 [1.03], *p* = .27 95% CI -2.81 to 0.80).

### Information processing speed and executive function (TMT AB)

The findings revealed a statistically significant effect of time, specifically a lower in TMT A scores for both groups (T3; *p* < .001) from T2 to T3. The findings revealed a statistically significant improvement in the TMT A scores of both groups from T2 to T3. Post-hoc analysis revealed that TMT A scores were significantly lower at T2 and T3 compared to T0 (Bonferroni-adjusted *p* < .001) for both groups. However, no significant effect at T1 (*p* = .04) after Bonferroni adjustment. No significant impact of group (*p* = .94) nor of group by time interaction (T3; *p* = .61) was observed on TMT A scores. At T1, LMM analysis showed no statistically significant difference in TMT A scores between the retrieval-only and EMD groups at T1 (M [ES] 37.90 [5.65] vs. 40.40 [5.73], *p* = .61, 95% CI -7.06 to 12.03), or at T2 (M [ES] 32.10 [5.65] vs. 35.00 [5.73], *p* = .55, 95% CI -6.68 to 12.41), or at T3 (M [ES] 27.00 [5.65] vs. 30.00 [5.73], *p* = .53, 95% CI -6.54 to 12.54) (Table 2).

Similarly, we found significant an overall effect of time for TMT B scores in both groups (appendix B). The results indicated a significant improvement in TMT B score from baseline to post-treatment at 3-months (*p* < .001) in both groups. Post-hoc analysis revealed that TMT B scores were lower at T2, and T3 than T0 for both groups (Bonferroni-adjusted *p* < .001), while again no significant effect was found at T1 after Bonferroni adjustment (*p =* .01). Thus, there was no effect of group (*p* = .21) nor of group by time interaction (T3; *p* = .58) on TMT B scores. LMM analysis showed no significant difference in TMT B scores between EMD group vs. retrieval-only at T1 (M [ES] 66.70 [8.14] vs. 68.00 [8.25], *p* = .84, 95% CI -11.97 to 14.59), or at T2 (M [ES] 56.50 [8.14] vs. 65.60 [8.25], *p* = .18, 95% CI -4.19 to 22.37) or at T3 (M [ES] 47.50 [8.14] vs. 52.20 [8.25], *p* = .49, 95% CI -8.62 to 17.94) (Table 2). Per-protocol analysis for TMT A and TMT B indicated similar results (appendix C).

### Attention/working memory (WAIS-IV-ID Digit Span)

There was an overall effect of time on the total score of Digit Span. The findings revealed that the Digit Span total score for both groups increased significantly from baseline to T3 (*p* < .001) (see appendix B). Post-hoc analysis indicated that the total scores for digit span were greater at T1, T2, and T3 compared to T0 (Bonferroni-adjusted *p* < .001) for both groups. However, neither the group (*p* = .64) nor the interaction of group by time (T3; *p* = .78) had any significant impact on the digit span scores. At T1, LMM analysis revealed no significant differences between the EMD and retrieval-only groups in terms of digit span total scores. (M [SE] 17.60 [0.72] vs. 18.40 [0.73], *p* = .15, 95% -0.30 to 1.97) or at T2 (M [SE] 18.30 [0.72] vs. 18.70 [0.73], *p* = .51, 95% CI -0.76 to 1.52) or at T3 (M [SE] 18.60 [0.72] vs. 18.20 [0.73], *p* = .47, 95% CI -1.55 to 0.72) (Table 2). Per-protocol analysis for the Digit Span total score indicated similar results (appendix C).

### Exploratory analyses

We conducted exploratory analyses to determine whether there were differences in neurocognitive outcomes when comparing recovered versus non-recovered patients and responders versus non-responders at T1, T2 and T3. At T1, in the retrieval-only group, 27 (67.5%) patients were classified as recovered, as compared to 13 (32.5%) non-recovered, and 24 (60%) were classified as responders, as compared to 16 (40%) non-responders. In addition, in the EMD group, 30 (68.2%) patients were classified as recovered, as compared to 14 (31.8%) non-recovered patients, and 30 (68.2%) patients were classified as responders, as compared to 14 (31.8%) non-responders.

We combined the data from EMD and retrieval-only groups because the groups did not differ significantly in terms of improvements in PTSD symptoms and neurocognitive outcomes over time. We calculated change scores (pre- minus post-treatment scores for all neurocognitive outcomes. We found no difference on CVLT, TMT AB and digit span scores between recovered as compared to non-recovered patients at T1 or T3. Likewise, there was no significant difference between responders as compared to non-responders on TMT AB and digit span scores at T1 or T3. However, at T2 we found a significant difference on CVLT total scores between responders as compared to non-responders and recovered as compared to non-recovered patients (*p* = .02). At T2, responders showed a better improvement than non-responding patients in CVLT total scores at T2 (M[SD]; -19.38 [16.47] vs. -11.10 [11.30], 95% CI 1.53 to 15.04, *p* = .02). Similarly, at T2 recovered patients showed a better improvement than non-recovered patients in CVLT total scores (M[SD]; -18.58 [15.59] vs. -11.28 [13.39], 95% CI 0.14 to 14.44, *p* = .02).

In addition, Appendix E and F present respectively, RCI’s participants’ cognitive tasks and reliability test retest of neurocognitive outcomes. The performance of cognitive tasks following treatment was likely to be statistically significant (RCI > 1.96), which could suggest clinically meaningful change, particularly at T1. Nevertheless, we discovered moderate test reliability for CVLT trial AB total (*r* = .58), TMT B (*r* = .50), and digit span (*r* = .58), indicating that we should interpret the finding with caution. The practice effect could potentially explain the performance improvement. Appendix G presents the number of participants showing impairment on each of the tests according to age- and education-corrected norm values at each time point T0, T1, T2, and T3. The results showed that the number of participants’ performances showing impairment decreased for each of the test from T0 to T3 following treatment for both groups.

## Discussion

The aim of the current study was to examine the effectiveness of EMD in improving neurocognitive functioning compared to a retrieval-only condition in patients with PTSD. Our study revealed no significant differences between the EMD and retrieval-only groups in neurocognitive outcomes at any time point. Thus, our findings did not support the hypothesis that EMs would be associated with a larger improvement in neurocognitive functioning than retrieval-only.

Instead, both EMD and retrieval-only conditions improved in terms of neurocognitive functioning from baseline to 3-month follow-up. However, since we did not employ alternative tests in our repeated assessments, the results of this analysis must be interpreted with caution, as they may include practice effects. Further, exploratory analyses suggested that treatment response and recovery in terms of PTSD symptoms, irrespective of the treatment condition, was associated with greater improvements in verbal learning as assessed with the CVLT as compared to non-responders and non-recovered patients at T2, respectively. However, no significant differences were found when comparing responders with non-responders and recovered with non-recovered patients on other neurocognitive outcomes including memory, attention, executive function, and information processing speed directly following or at 3 months after the intervention.

Our current results did not support the hypothesis EMs enhance neurocognitive outcomes. This consistent with the finding of Lee and Cuijpers (2013) [38], who discovered that while eye movements may reduce emotional distress, their impact on cognitive functions is minimal. Additionally, recent neuroimaging studies [77] suggest that alterations in brain activation patterns link to the treatment of PTSD are not necessarily tied to the use of eye movements.

Our findings suggest that using EMs in EMDR may not provide any further cognitive advantages compare to retrieval-only in clinical samples. The results are in line with previous studies [78, 79], which similarly reported no substantial evidence supporting the unique contribution of eye movement in enhancing cognitive function during therapy for PTSD. Our study results provide further evidence that there is no difference between EMDR with and without EMs, nor do EMs add important value to the EMDR therapy process, at least not in clinical samples. The scarcity of trial studies conducted in the current study has been a major obstacle to believing in the additional contribution of EMs in EMDR [80].

The results of our study do not support the working memory theory of EMDR, which posits that eye movements during memory recall tax the working memory, therefore diminishing the vividness and emotional impact of traumatic memory [35]. Analogue experimental studies involving healthy subjects support the working memory model. These analogue studies found that performing dual tasks such as EMs during retrieval significantly reduced the vividness and emotionality of these aversive memories, compared to retrieval-only [39]. However, these studies were not carried out among PTSD patients, and no clinical or neurocognitive outcomes were assessed. Note that we also did not find any differences between study arms in clinical outcomes, or in terms of emotionality and vividness of the target memory in the same study population [56].

The results of our exploratory analysis strengthen the evidence that responding or recovered patients appeared to improve particularly in terms of verbal memory (i.e., word-list memory and learning strategies). The finding is consistent with our recent systematic review examining the effects of psychological interventions on neurocognitive functioning PTSD [21] that found that memory outcomes were the only neurocognitive function that improved following effective PTSD treatment. Psychotherapy relies significantly on neurocognitive skills to accomplish treatment benefits via cognitive restructuring, association updating, and behavioural modifications [26]. Additionally, findings from the PTSD treatment literature suggest that effective PTSD treatment may enhance neurocognitive skills and its indirect effects on risk and resilience factors such as coping [25]. The neuroimaging literature suggests that these improvements in neurocognitive functioning are accompanied by increased activation in cognitive control networks (prefrontal cortex), decreased amygdala activation, and increased connectivity between amygdala and prefrontal cortex [25].

### Strengths and limitations

To the best of our knowledge, this is the first RCT focusing on the potential effects of EMs in EMD on neurocognitive functioning in Indonesian PTSD patients. A particular strength is the dismantling design, in which we compared a singular element within a complex EMDR treatment. The study comprised participants who were diagnosed with PTSD using the SCID-5 scale. The dropout rate was relatively low, suggesting a high level of participant retention. The low dropout rate can enhance the statistical power of the current study.

However, the present study also had several limitations. Firstly, the study may have been underpowered to detect smaller but clinically meaningful differences in neurocognitive outcomes between the two treatment conditions, despite the fact that we achieved our target sample size. Six participants also did not complete neurocognitive assessment at T3 because of Covid-19 situational restrictions, and these measures could not be administered online. Second, the objective performance validity was not performed, as we relied solely on adapted and validated tests in the same the language from other studies. Given that PTSD is known to be associated with higher rates of invalid test performance on neuropsychological tests, this could potentially impact the credibility and generalizability of the study. Third, we used the same form tests repeatedly at T1, T2, and T3, which can lead to an increase in T2 and T3 scores as a practice effect. The alternate test form is needed to minimize practice effect. In addition, there is variability in therapist expertise that allows for differences in therapy results. Fourth, the predominantly female sample (91.2%) restrict the generalizability of the findings to male PTSD patients. Additionally, the study was conducted in Indonesia, and cultural differences in trauma processing and treatment response could restrict the applicability of the finding to other populations.

### Clinical implications

In clinical practice, trauma-focused treatments such as EMDR and other retrieval-based PTSD treatments should continue to be utilized for PTSD treatment, as both approaches appear to improve neurocognitive functioning. Clinicians should consider these findings when designing treatment plans, focusing on therapeutic techniques that facilitate memory retrieval and cognitive restructuring. Clinicians should be aware that the addition of eye movements may not enhance cognitive outcomes. Consequently, they may opt for simpler retrieval-based approaches without compromising cognitive outcomes. In terms of assessing outcomes of PTSD treatment, the neurocognitive functioning assessment may be a valuable additional metric to evaluate the effectiveness of treatments as well, especially a measure for verbal learning and memory such as the CVLT. In addition, the finding that verbal memory performance is particularly improves along with positive treatment response, may provide additional motivation for patients with PTSD to pursue effective treatment.

### Research implications

Recommendations for future research on EMDR include the recruitment of more varied and larger clinical samples to validate the finding and enhance the generalizability. Future research may further investigate the specific components of trauma-focused therapies, including EMDR, that contribute to cognitive improvements and explore innovative methods to enhance these effects. Ideally, these studies should have larger sample size to allow sufficient statistical power. In addition, in future RCTs researchers should align the instruments they use to assess neurocognitive outcomes and make their data available to other researcher for individual data patient meta-analysis. This would allow datasets to be combined, leading to more statistical power to detect subtle mechanistic effects. Furthermore, the use of objective measures when administering bilateral EMs stimulation (e.g., eye-tracking software) may give a better understanding of the type of EMs that take place and their impact on the efficacy of treatment. The use of these measures may also add information about physiological changes, thus generating more information about the neurobiological foundation underlying EMs mechanisms in EMDR.

## Conclusion

Our study did not find evidence to support the notion that the EMs component in EMDR significantly enhances neurocognitive functioning compared to a retrieval-only condition. Both EMD and retrieval-only treatments were associated with improvements in verbal memory, working memory, executive function, and information processing speed, implying that therapeutic benefits of these interventions may not be dependent on the inclusion of eye movements. We conclude that treatments based on retrieval of traumatic memories can be used to improve neurocognitive functioning in patients with PTSD, who are known to often experience memory and attention deficits.

## Electronic supplementary material

Below is the link to the electronic supplementary material.

Supplementary Material 1

Supplementary Material 2

Supplementary Material 3

Supplementary Material 4

Supplementary Material 5

Supplementary Material 6

Supplementary Material 7

## Data Availability

The data that supports the findings of this study are available on request from the corresponding author. The data is not publicly available due to privacy or ethical restrictions.

## Abbreviations

PTSD: Posttraumatic stress disorder
DSM-5: The Diagnostic and Statistical Manual of Mental Disorders Fifth Edition
CVLT: California Verbal Learning Test
RCTs: Randomized controlled trials
EMDR: Eye movement desensitization and reprocessing
EMD: Eye movement desensitization
BEP: Brief Eclectic Psychotherapy
EMs: Eye movements
PCL-5: PTSD Checklist for DSM-5
SCID-5: Structured Clinical Interview for DSM-5 disorders
HRV: Heart rate variability
TMT: Trail Making Test TMT
CVLT: California Learning Verbal Test
WAIS-IV: Wechsler Adult Intelligence Scale fourth edition
SUD: Subjective Units of Distress
ITT: Intent to treat
LMM: Linear Mixed Models
