# Supplementary material for: The effect of eye movement desensitization on neurocognitive functioning compared to retrieval-only in PTSD patients: a randomized controlled trial
Source: BMC Psychiatry. 2024 Dec 27;24:956. doi: 10.1186/s12888-024-06420-9 (PMC11673372; doi:10.1186/s12888-024-06420-9)
Supplement: Supplementary file 2 — Supplementary Material 2 [file 12888_2024_6420_MOESM2_ESM.docx]

Appendix B Estimates for the effect of group, time, and group-time interaction (intention to treat, N = 91)

| **Variable** | **Time** | | | | | | | | | **Time-group interaction** | | | | | | | | |
| --- | --- | --- | --- | --- | --- | --- | --- | --- | --- | --- | --- | --- | --- | --- | --- | --- | --- | --- |
|  | **T1** | | | **T2** | | | **T3** | | | **T1** | | | **T2** | | | **T3** | | |
|  | **β** | **SE** | ***p*-val** | **β** | **SE** | ***p*-val** | **β** | **SE** | ***p*-val** | **β** | **SE** | ***p*-val** | **β** | **SE** | ***p*-val** | **β** | **SE** | ***p*-val** |
| **CVLT** | | | | | | | | | | | | | | | | | | |
| CVLT total | 12.00 | 1.83 | 0.00 | 17.54 | 1.83 | 0.00 | 20.89 | 1.83 | 0.00 | -1.05 | 2.55 | 0.68 | -0.93 | 2.55 | 0.72 | 0.11 | 2.55 | 0.97 |
| Trial A | 7.20 | 1.25 | 0.00 | 10.56 | 1.25 | 0.00 | 12.55 | 1.25 | 0.00 | -0.49 | 1.74 | 0.78 | 0.04 | 1.74 | 0.98 | 1.65 | 1.74 | 0.34 |
| Trial B | 0.63 | 0.31 | 0.04 | 0.77 | 0.31 | 0.01 | 1.26 | 0.31 | 0.00 | -072 | 0.43 | 0.09 | -061 | 0.43 | 0.15 | -0.45 | 0.43 | 0.29 |
| Delay A | 4.35 | 0.77 | 0.00 | 4.69 | 0.77 | 0.00 | 5.20 | 0.77 | 0.00 | -1.06 | 1.07 | 0.32 | 0.34 | 1.07 | 0.75 | 0.33 | 1.07 | 0.76 |
| **TMT** |  |  |  |  |  |  |  |  |  |  |  |  |  |  |  |  |  |  |
| TMT A | -7.54 | 3.73 | 0.04 | -12.95 | 3.73 | 0.00 | -17.95 | 3.73 | 0.00 | -2.11 | 5.19 | 0.68 | -2.49 | 5.19 | 0.63 | -2.63 | 5.19 | 0.61 |
| TMT B | -12.80 | 4.82 | 0.01 | -15.21 | 4.82 | 0.00 | -28.60 | 4.82 | 0.00 | 7.07 | 6.70 | 0.29 | -0.70 | 6.70 | 0.92 | 3.73 | 6.70 | 0.58 |
| **Digit Span** |  |  |  |  |  |  |  |  |  |  |  |  |  |  |  |  |  |  |
| Digit Span total | 1.76 | 0.38 | 0.00 | 1.98 | 0.38 | 0.00 | 1.56 | 0.38 | 0.00 | -1.10 | 0.53 | 0.04 | -0.65 | 0.53 | 0.22 | 0.15 | 0.53 | 0.78 |
| Forward | 0.59 | 0.20 | 0.00 | 0.73 | 0.20 | 0.00 | 0.57 | 0.20 | 0.00 | -0.36 | 0.28 | 0.20 | -0.41 | 0.28 | 0.14 | -0.38 | 0.28 | 0.17 |
| Backward | 0.46 | 0.18 | 0.01 | 0.41 | 0.18 | 0.02 | 0.36 | 0.18 | 0.04 | -0.26 | 0.25 | 0.29 | 0.21 | 0.25 | 0.40 | 0.06 | 0.25 | 0.80 |
| Sequence | 0.59 | 0.22 | 0.01 | 0.89 | 0.22 | 0.00 | 0.93 | 0.22 | 0.00 | -0.27 | 0.30 | 0.37 | -0.31 | 0.30 | 0.30 | 0.07 | 0.30 | 0.82 |

Notes:

SE = Standard error, CVLT = the California Verbal Learning Test, TMT = Trail Making Test, T1 = time point at a week after treatment session, T2 = time point at one month after treatment session , T3 = 3-month after treatment session
