# Supplementary material for: The effect of eye movement desensitization on neurocognitive functioning compared to retrieval-only in PTSD patients: a randomized controlled trial
Source: BMC Psychiatry. 2024 Dec 27;24:956. doi: 10.1186/s12888-024-06420-9 (PMC11673372; doi:10.1186/s12888-024-06420-9)
Supplement: Supplementary file 3 — Supplementary Material 3 [file 12888_2024_6420_MOESM3_ESM.docx]

Appendix C. Estimate for the effect of group, time, and time- group interaction (per protocol, N (T1)= 84, N (T2)= 82, N (T3) = 63)

| **Variable** | **Time** | | | | | | | | | **Time-group interaction** | | | | | | | | |  |
| --- | --- | --- | --- | --- | --- | --- | --- | --- | --- | --- | --- | --- | --- | --- | --- | --- | --- | --- | --- |
|  | **T1** | | | **T2** | | | **T3** | | | **T1** | | | **T2** | | | **T3** | | | |
|  | **β** | **SE** | ***p*-val** | **β** | **SE** | ***p*-val** | **β** | **SE** | ***p*-val** | **β** | **SE** | ***p*-val** | **β** | **SE** | ***p*-val** | **β** | **SE** | ***p*-val** | |
| **CVLT** |  |  |  |  |  |  |  |  |  |  |  |  |  |  |  |  |  |  | |
| CVLT total | 11.46 | 2.11 | 0.00 | 15.91 | 2.11 | 0.00 | 19.07 | 2.11 | 0.00 | 0.35 | 3.03 | 0.91 | 1.93 | 3.03 | 0.53 | 1.03 | 3.03 | 0.74 | |
| Trial A | 7.15 | 1.39 | 0.00 | 11.09 | 1.39 | 0.00 | 12.66 | 1.39 | 0.00 | 0.88 | 1.99 | 0.66 | 1.55 | 1.99 | 0.44 | 1.57 | 1.99 | o.43 | |
| Trial B | 0.49 | 0.34 | 0.15 | 0.61 | 0.34 | 0.08 | 1.21 | 0.34 | 0.00 | -0.58 | 0.49 | 0.23 | -0.28 | 0.49 | 0.56 | -0.53 | 0.49 | 0.28 | |
| Delay A | 3.82 | 0.91 | 0.00 | 4.21 | 0.91 | 0.00 | 4.95 | 0.91 | 0.00 | 0.05 | 1.31 | 0.97 | 0.66 | 1.31 | 0.62 | 0.25 | 1.31 | 0.85 | |
| **TMT** |  |  |  |  |  |  |  |  |  |  |  |  |  |  |  |  |  |  | |
| TMT A | -5.88 | 3.34 | 0.08 | -11.06 | 3.34 | 0.00 | -12.87 | 3.34 | 0.00 | -1.06 | 4.80 | 0.83 | -1.10 | 4.80 | 0.82 | -2.77 | 4.80 | 0.57 | |
| TMT B | -12.06 | 5.11 | 0.02 | -12.42 | 5.11 | 0.02 | -23.81 | 5.11 | 0.00 | 3.87 | 7.34 | 0.60 | 0.04 | 7.34 | 0.99 | 0.55 | 7.34 | 0.94 | |
| **Digit Span** |  |  |  |  |  |  |  |  |  |  |  |  |  |  |  |  |  |  | |
| Digit Span total | 1.94 | 0.44 | 0.00 | 1.94 | 0.44 | 0.00 | 1.80 | 0.44 | 0.00 | -1.39 | 0.63 | 0.03 | -0.52 | 0.63 | 0.41 | -0.29 | 0.63 | 0.65 | |
| Forward | 0.58 | 0.23 | 0.01 | 0.67 | 0.23 | 0.00 | 0.52 | 0.23 | 0.02 | -0.41 | 0.32 | 0.20 | -0.31 | 0.32 | 0.34 | -0.42 | 0.32 | 0.20 | |
| Backward | 0.70 | 0.20 | 0.00 | 0.39 | 0.20 | 0.05 | 0.55 | 0.20 | 0.01 | -0.57 | 0.29 | 0.05 | 0.19 | 0.29 | 0.52 | 0.07 | 0.29 | 0.82 | |
| Sequence | 0.67 | 0.25 | 0.01 | 0.88 | 0.25 | 0.00 | 0.70 | 0.25 | 0.01 | -0.41 | 0.35 | 0.25 | -0.40 | 0.35 | 0.27 | 0.11 | 0.35 | 0.76 | |

Notes:

SE = Standard error, CVLT= the California Verbal Learning test, TMT = Trail Making Test , T1 = time point at a week after treatment session, T2 = time point at one month after treatment session , T3 = 3-month after treatment session.
