# Supplementary material for: The effect of eye movement desensitization on neurocognitive functioning compared to retrieval-only in PTSD patients: a randomized controlled trial
Source: BMC Psychiatry. 2024 Dec 27;24:956. doi: 10.1186/s12888-024-06420-9 (PMC11673372; doi:10.1186/s12888-024-06420-9)
Supplement: Supplementary file 4 — Supplementary Material 4 [file 12888_2024_6420_MOESM4_ESM.docx]

D. Summary statistics and results from mixed-model analysis of CVLT, TMT and Digit Span (Per protocol, N(T1)=84, N (T2) =82, N (T3) =63)

| **Outcomes** | **Measurement time** | **Mean (Standard Error)** | | **Mean difference (95% confidence interval)** | **p-value** |
| --- | --- | --- | --- | --- | --- |
|  |  | **Retrieval-only** | **EMD** |  |  |
| CVLT total | T1 | 88.2 (3.29) | 90,1 (3,31) | -1.85 (-7.87 to 4.17) | 0.54 |
|  | T2 | 92.7 (3.29) | 96,1 (3,31) | -3.43 (-9.45 to 2.59) | 0.26 |
|  | T3 | 95.8 (3.29) | 98,1 (3,31) | -2.53 (-8.55 to 3.49) | 0.40 |
| CVLT trial A | T1 | 56.7 (2.11) | 57.9 (2.12) | -1.21 (-5.09 to 2.67) | 0.53 |
|  | T2 | 60.6 (2.11) | 62.5 (2.12) | -1.87 (-5.77 to 1.99) | 0.33 |
|  | T3 | 62.2 (2.11) | 64.1 (2.12) | -1.90 ( -5.78 to 1.98) | 0.33 |
| CVLT trial B | T1 | 6.51 (0.45) | 6.29 (0.46) | 0.22 (-0.64 to 1.10) | 0.61 |
|  | T2 | 6.63 (0.45) | 6.70 (0.46) | -0.07 (-0.94 to 0.80) | 0.87 |
|  | T3 | 7.23 (0.45) | 7.06 (0.46) | 0.17 (-0.70 to 1.04) | 0.69 |
| CVLT delayed | T1 | 25.1 (1.14) | 25.9 (1.15) | -0.87 (-3.11 to 1.38) | 0.44 |
|  | T2 | 15.5 (1.14) | 26.9 (1.15) | -1.47 (-3.71 to 0,77) | 0.19 |
|  | T3 | 26.2 (1.14) | 27.3 (1.15) | -1.06 (-3.30 to 1.18) | 0.35 |
| TMT A | T1 | 40.0 (5.22) | 40.7 (5.26) | -0.69 (-10.24 to 8.85) | 0.88 |
|  | T2 | 34.8 (5.22) | 35.5 (5.26) | -0.65 (-10.19 to 8.90) | 0.89 |
|  | T3 | 33.0 (5.22) | 32.00 (5.26) | 1.02 (-8.52 to 10.57) | 0.83 |
| TMT B | T1 | 67.0 (8.05) | 66.8 (8.12) | 0.23 (-14.48 to 14.93) | 0.98 |
|  | T2 | 66.7 (8.05) | 62.6 (8.12) | 4.06 (-10.65 to 18.76) | 0.58 |
|  | T3 | 55.3 (8.05) | 51.7 (8.12) | 3.54 (-11.17 to 18.25) | 0.63 |
| Digit span total | T1 | 18.3 (0.79) | 17.6 (0.8) | 0.71 (-0.69 to 2.11) | 0.31 |
|  | T2 | 18.3 (0.79) | 18.5 (0.8) | -0.16 (-1.56 to 1.24) | 0.82 |
|  | T3 | 18.2 (0.79) | 18.6 (0.8) | -0.39 (-1.79 to 1.01) | 0.58 |
| Digit span forward | T1 | 6.21 (0.32) | 6.28 (0.32) | -0.07 (-0.67 to 0.53) | 0.81 |
|  | T2 | 6.30 (0.32) | 6.48 (0.32) | -0.17 (-0.78 to 0.42) | 0.56 |
|  | T3 | 6.15 (0.32) | 6.22 (0.32) | -0.07 (-0.67 to 0.53) | 0.22 |
| Digit span backward | T1 | 5.02 (0.30) | 4.67 (0.31) | 0.35 (-0.21 to 0.91) | 0.22 |
|  | T2 | 4.72 (0.30) | 5.13 (0.31) | -0.40 (-0.97 to 0.16) | 0.15 |
|  | T3 | 4.87 (0.30) | 5.16 (0.31) | -0.28 (-0.85 to 0.28) | 0.31 |
| Digit span sequence | T1 | 7.09 (0.35) | 6.66 (0.35) | 0.43 (-0.22 to 1.09)) | 0.19 |
|  | T2 | 7.3 (0.35) | 6.88 (0.35) | 0.42 (-0.24 to 1.08) | 0.20 |
|  | T3 | 7.12 (0.35) | 7.20 (0.35) | -0.08 (-0.74 to 0.57)) | 0.80 |

Notes:

*Bonferroni correction-significant, *p* < .005

EMD = Eye Movement Desensitization

CVLT; the California Verbal Learning, PTSD; Posttraumatic Stress Disorder, TMT; Trail Making Test
