# Supplementary material for: The effect of eye movement desensitization on neurocognitive functioning compared to retrieval-only in PTSD patients: a randomized controlled trial
Source: BMC Psychiatry. 2024 Dec 27;24:956. doi: 10.1186/s12888-024-06420-9 (PMC11673372; doi:10.1186/s12888-024-06420-9)
Supplement: Supplementary file 5 — Supplementary Material 5 [file 12888_2024_6420_MOESM5_ESM.docx]

| Appendix E. Percentage of reliable change index (RCIs) based on a 95% confidence level   \|  \| \| \| \| \| \|  \|  \|  \|  \|  \|  \|  \|  \|  \| \| --- \| --- \| --- \| --- \| --- \| --- \| --- \| --- \| --- \| --- \| --- \| --- \| --- \| --- \| --- \| \| Test \| Higher \| \| \| Lower \| \| \|  \|  \|  \|  \|  \|  \|  \|  \| \| T1 \| T2 \| T3 \| T1 \| T2 \| T3 \|  \|  \|  \|  \|  \|  \|  \|  \| \| CVLT \|  \|  \|  \|  \|  \|  \|  \|  \|  \|  \|  \|  \|  \|  \| \| Trial A \| 4.5 \| 0.0 \| 3.2 \| 0.0 \| 0.0 \| 3.2 \|  \|  \|  \|  \|  \|  \|  \|  \| \| Trial B \| 0.0 \| 0.0 \| 0.0 \| 2.3 \| 2.3 \| 0.0 \|  \|  \|  \|  \|  \|  \|  \|  \| \| Delay A \| 2.3 \| 0.0 \| 3.2 \| 0.0 \| 0.0 \| 0.0 \|  \|  \|  \|  \|  \|  \|  \|  \| \| CVLT total (trial AB) \| 4.5 \| 0.0 \| 3.2 \| 2.3 \| 0.0 \| 3.2 \|  \|  \|  \|  \|  \|  \|  \|  \| \| TMT \|  \|  \|  \|  \|  \|  \|  \|  \|  \|  \|  \|  \|  \|  \| \| TMT A \| 2.3 \| 4.7 \| 0.0 \| 2.3 \| 2.3 \| 0.0 \|  \|  \|  \|  \|  \|  \|  \|  \| \| TMT B \| 4.5 \| 2.3 \| 0.0 \| 0.0 \| 4.7 \| 6.5 \|  \|  \|  \|  \|  \|  \|  \|  \| \| Digit Span \|  \|  \|  \|  \|  \|  \|  \|  \|  \|  \|  \|  \|  \|  \| \| Forward \| 4.5 \| 4.7 \| 0.0 \| 6.8 \| 0.0 \| 3.2 \|  \|  \|  \|  \|  \|  \|  \|  \| \| Backward \| 2.3 \| 0.0 \| 3.2 \| 4.5 \| 0.0 \| 3.2 \|  \|  \|  \|  \|  \|  \|  \|  \| \| Sequence \| 2.3 \| 7.0 \| 9.7 \| 4.5 \| 0.0 \| 0.0 \|  \|  \|  \|  \|  \|  \|  \|  \| \| Digit Span total \| 2.3 \| 7.0 \| 0.0 \| 11.4 \| 0.0 \| 0.0 \|  \|  \|  \|  \|  \|  \|  \|  \| \| Reference: \|  \|  \|  \|  \|  \|  \|  \|  \|  \|  \|  \|  \|  \|  \| \| Maassen. G. H.. Bossema. E.. & Brand. N. (2009). Reliable change and practice effects:  Outcomes of various indices compared. *Journal of clinical and experimental neuropsychology* . *31* (3). 339-352.  Appendix F. Test re-test scores of neurocognitive outcomes (Pearson correlation) \| \| \| \| \| \| \| \| \| \| \| \| \| \| \| | | | | | | | | | | | | | | |  |  |  |  |  |  |  |  |  |
| --- | --- | --- | --- | --- | --- | --- | --- | --- | --- | --- | --- | --- | --- | --- | --- | --- | --- | --- | --- | --- | --- | --- | --- | --- | --- | --- | --- | --- | --- | --- | --- | --- | --- | --- | --- | --- | --- | --- | --- | --- | --- | --- | --- | --- | --- | --- | --- | --- | --- | --- | --- | --- | --- | --- | --- | --- | --- | --- | --- | --- | --- | --- | --- | --- | --- | --- | --- | --- | --- | --- | --- | --- | --- | --- | --- | --- | --- | --- | --- | --- | --- | --- | --- | --- | --- | --- | --- | --- | --- | --- | --- | --- | --- | --- | --- | --- | --- | --- | --- | --- | --- | --- | --- | --- | --- | --- | --- | --- | --- | --- | --- | --- | --- | --- | --- | --- | --- | --- | --- | --- | --- | --- | --- | --- | --- | --- | --- | --- | --- | --- | --- | --- | --- | --- | --- | --- | --- | --- | --- | --- | --- | --- | --- | --- | --- | --- | --- | --- | --- | --- | --- | --- | --- | --- | --- | --- | --- | --- | --- | --- | --- | --- | --- | --- | --- | --- | --- | --- | --- | --- | --- | --- | --- | --- | --- | --- | --- | --- | --- | --- | --- | --- | --- | --- | --- | --- | --- | --- | --- | --- | --- | --- | --- | --- | --- | --- | --- | --- | --- | --- | --- | --- | --- | --- | --- | --- | --- | --- | --- | --- | --- | --- | --- | --- | --- | --- | --- | --- | --- | --- | --- | --- | --- | --- | --- | --- | --- | --- | --- | --- | --- | --- | --- | --- | --- | --- | --- | --- | --- | --- | --- | --- | --- | --- | --- | --- | --- | --- | --- | --- | --- | --- | --- | --- | --- | --- | --- | --- | --- | --- | --- | --- | --- | --- | --- | --- | --- | --- | --- | --- | --- | --- | --- | --- | --- | --- | --- | --- | --- | --- | --- | --- | --- | --- | --- | --- | --- | --- | --- | --- | --- | --- |
| Pearson’s correlation coefficient (Pearson’s r) | | | | | | | |  |  |  |  |  |  |  |  |  |  |  |  |  |  |  |  |
| Test | T1 | T2 | T3 |  |  |  |  |  |  |  |  |  |  |  |  |  |  |  |  |  |  |  |  |
| CVLT |  |  |  |  |  |  |  |  |  |  |  |  |  |  |  |  |  |  |  |  |  |  |  |
| Trial A | 0.60 | 0.34 | 0.44 |  |  |  |  |  |  |  |  |  |  |  |  |  |  |  |  |  |  |  |  |
| Trial B | 0.16 | 0.31 | 0.50 |  |  |  |  |  |  |  |  |  |  |  |  |  |  |  |  |  |  |  |  |
| Delay A | 0.34 | 0.13 | 0.38 |  |  |  |  |  |  |  |  |  |  |  |  |  |  |  |  |  |  |  |  |
| CVLT total (trial AB) | 0.58 | 0.37 | 0.46 |  |  |  |  |  |  |  |  |  |  |  |  |  |  |  |  |  |  |  |  |
| TMT |  |  |  |  |  |  |  |  |  |  |  |  |  |  |  |  |  |  |  |  |  |  |  |
| TMT A | 0.43 | 0.45 | 0.44 |  |  |  |  |  |  |  |  |  |  |  |  |  |  |  |  |  |  |  |  |
| TMT B | 0.50 | 0.63 | 0.51 |  |  |  |  |  |  |  |  |  |  |  |  |  |  |  |  |  |  |  |  |
| Digit Span |  |  |  |  |  |  |  |  |  |  |  |  |  |  |  |  |  |  |  |  |  |  |  |
| Forward | 0.45 | 0.39 | 0.31 |  |  |  |  |  |  |  |  |  |  |  |  |  |  |  |  |  |  |  |  |
| Backward | 0.48 | 0.37 | 0.28 |  |  |  |  |  |  |  |  |  |  |  |  |  |  |  |  |  |  |  |  |
| Sequence | 0.45 | 0.41 | 0.30 |  |  |  |  |  |  |  |  |  |  |  |  |  |  |  |  |  |  |  |  |
| Digit Span total | 0.58 | 0.53 | 0.45 |  |  |  |  |  |  |  |  |  |  |  |  |  |  |  |  |  |  |  |  |
| Reference: |  |  |  |  |  |  |  |  |  |  |  |  |  |  |  |  |  |  |  |  |  |  |  |

Karlsen. R. H.. Karr. J. E.. Saksvik. S. B.. Lundervold. A. J.. Hjemdal. O.. Olsen. A.. ... & Skandsen. T. (2022). Examining 3-month test-retest reliability and reliable change using the Cambridge Neuropsychological Test Automated Battery. *Applied Neuropsychology: Adult*. *29*(2). 146-154.
