# Supplementary material for: The effect of eye movement desensitization on neurocognitive functioning compared to retrieval-only in PTSD patients: a randomized controlled trial
Source: BMC Psychiatry. 2024 Dec 27;24:956. doi: 10.1186/s12888-024-06420-9 (PMC11673372; doi:10.1186/s12888-024-06420-9)
Supplement: Supplementary file 6 — Supplementary Material 6 [file 12888_2024_6420_MOESM6_ESM.docx]

Appendix G. The number of participants showing impairment at each measurement time point T0, T1, T2, and T3 as follows:

| Measures (cutoff) | Retrieval-only (n^c^) | | | | EMD (n^c^) | | | |
| --- | --- | --- | --- | --- | --- | --- | --- | --- |
|  | T0 | T1 | T2 | T3 | T0 | T1 | T2 | T3 |
|  | n (%) | n (%) | n (%) | n (%) | n (%) | n (%) | n (%) | n (%) |
| CVLT^a^ | | | | | | | | |
| Trial A (<43) | 11 (25) | 1 (3) | 1 (3) | 0 (0) | 4 (9) | 1 (2) | 0 (0) | 1 (3) |
| Delay A (<8) | 1 (2) | 0 (0) | 1 (3) | 0 (0) | 1 (2) | 0 (0) | 0 (0) | 0 (0) |
| TMT^a^ | | | | | | | | |
| TMT A (>48”) | 16 (36) | 6 (15) | 6 (15) | 2 (6) | 16 (34) | 9 (20) | 6 (14) | 3 (10) |
| TMT B (>117”) | 4 (9) | 4 (10) | 3 (8) | 1 (3) | 4 (9) | 5 (11) | 2 (5) | 0 (0) |
| Digit Span^b^ | | | | | | | | |
| Forward (<6) | 20 (45) | 11 (28) | 9 (23) | 8 (25) | 15 (32) | 13 (3) | 8 (19) | 7 (23) |
| Backward (<5) | 23 (52) | 13 (33) | 14 (36) | 11 (34) | 26 (55) | 22 (50) | 10 (23) | 9 (29) |
| Sequence (<5) | 6 (14) | 5 (13) | 2 (5) | 1 (3) | 6 (13) | 2 (5) | 3 (7) | 1 (3) |
| Total Digit Span  (<16) | 15 (34) | 11 (28) | 4 (10) | 4 (13) | 17 (36) | 11 (25) | 8 (19) | 3 (10) |

1. Nijdam, M.J., Gersons, B.P., & Olff, M. (2013). The role of major depression in neurocognitive functioning in patients with posttraumatic stress disorder. *European Journal of Psychotraumatology* , *4* (1), 19979.
2. Webber, T. A., & Soble, J. R. (2018). Utility of various WAIS-IV Digit Span indices for identifying noncredible performance validity among cognitively impaired and unimpaired examinees. *The Clinical Neuropsychologist*, *32*(4), 657-670.
3. Sample size (n): retrieval-only T0= 44, T1= 40, T2= 39, T3= 32; EMD T0= 47, T1= 44, T2= 43, T3= 31
