# Supplementary material for: The effect of eye movement desensitization on neurocognitive functioning compared to retrieval-only in PTSD patients: a randomized controlled trial
Source: BMC Psychiatry. 2024 Dec 27;24:956. doi: 10.1186/s12888-024-06420-9 (PMC11673372; doi:10.1186/s12888-024-06420-9)
Supplement: Supplementary file 7 — Supplementary Material 7 [file 12888_2024_6420_MOESM7_ESM.docx]

Appendix H. List of medications that were considered exclusionary of the study

| Schedule I | Schedule II |
| --- | --- |
| Drugs, substance, are defines as with no currently accepted medical use and a high potential for abuse | Drugs, substance, are defines as drugs with a high potential for abuse, which use potentially leading to severe psychological or physical dependence |
| Heroin  Lyserdic acid diethylamide (LSD)  Marijuana (cannabis)  3,4-methylenedioxymethamphetamine (ecstasy)  Methaqualone  Peyote | Combination products with lesaa than 15 mg of Hydrocodones per dosage unit (Vicodin)  Cocaine  Methamphetamine  Methadone  Hydromorphone (Dilaudid)  Meperidine (Demerol)  Oxycodone (OxyContin)  Fentanyl  Dexedrine  Adderall  Ritalin |

Reference:

DEA. 10 July, 2018. Drug Scheduling. Retrieved from https://www.dea.gov/drug-information/drug-scheduling on 2 October, 2024
